# Association of Hypokalemia Incidence and Better Treatment Response in NSCLC Patients: A Meta-Analysis and Systematic Review on Anti-EGFR Targeted Therapy Clinical Trials

**DOI:** 10.3389/fonc.2021.757456

**Published:** 2022-01-05

**Authors:** Jiawei Zhou, Jianling Bai, Yuanping Yue, Xin Chen, Theis Lange, Dongfang You, Yang Zhao

**Affiliations:** ^1^ Department of Biostatistics, School of Public Health, Nanjing Medical University, Nanjing, China; ^2^ Section of Biostatistics, Department of Public Health, Faculty of Health and Medical Sciences, University of Copenhagen, Copenhagen, Denmark; ^3^ Jiangsu Key Lab of Cancer Biomarkers, Prevention and Treatment, Collaborative Innovation Center for Cancer Medicine, Nanjing Medical University, Nanjing, China

**Keywords:** hypokalemia, targeted therapy, EGFR antagonist, NSCLC, meta-analysis

## Abstract

**Background:**

This meta-analysis was designed to explore the relationship between the level of serum potassium and the treatment effect of epidermal growth factor receptor (EGFR) antagonist in advanced non-small cell lung cancer (aNSCLC).

**Methods:**

We searched phase II/III prospective clinical trials on treatment with EGFR antagonists for aNSCLC patients. The objective response rate (ORR) and/or the disease control rate (DCR) and the incidence of hypokalemia of high grade (equal to or greater than grade 3) were summarized from all eligible trials. Heterogeneity, which was evaluated by Cochran’s *Q*-test and the *I* ^2^ statistics, was used to determine whether a random effects model or a fixed effects model will be used to calculate pooled proportions. Subgroup analysis was performed on different interventions, line types, phases, and drug numbers.

**Results:**

From 666 potentially relevant articles, 36 clinical trials with a total of 9,761 participants were included in this meta-analysis. The pooled ORR was 16.25% (95%CI = 12.45–21.19) when the incidence of hypokalemia was 0%–5%, and it increased to 34.58% (95%CI = 24.09–45.07) when the incidence of hypokalemia was greater than 5%. The pooled DCR were 56.03% (95%CI = 45.03–67.03) and 64.38% (95%CI = 48.60–80.17) when the incidence rates of hypokalemia were 0%–5% and greater than 5%, respectively. The results of the subgroup analysis were consistent with the results of the whole population, except for not first-line treatment, which may have been confounded by malnutrition or poor quality of life in long-term survival.

**Conclusion:**

The efficacy of anti-EGFR targeted therapy was positively associated with the hypokalemia incidence rate. Treatment effects on the different serum potassium strata need to be considered in future clinical trials with targeted therapy.

## Introduction

Lung cancer is the most common cause of death from cancer, accounting for 1.80 million deaths in 2020, and its incidence was still increasing (1, 2). Based on cell origin, non-small cell lung cancer (NSCLC) is responsible for 80%–85% of lung primary malignancies (3). As a transmembrane glycoprotein, epidermal growth factor receptor (EGFR) was the first growth factor receptor to be proposed as a target for cancer therapy (4). EGFR is a member of the ErbB family of receptors that, once activated, leads to the excitation of subsequent intracellular signaling pathways; it can regulate cellular proliferation, differentiation, migration, and apoptosis (5, 6). There are two main classes of EGFR antagonists: anti-EGFR monoclonal antibodies (e.g., cetuximab and panitumumab) and small-molecule EGFR tyrosine kinase inhibitors (TKIs) (e.g., erlotinib and gefitinib) (4). These antagonists exert their activities through binding to the extracellular domain of EGFR, competing for receptor binding by occluding the ligand-binding region, blocking the ligand-induced EGFR tyrosine kinase activation, and inhibiting EGFR autophosphorylation and downstream signaling (4, 7). EGFR antagonists are beneficial for human epithelial cancers, especially for lung carcinoma.

Potassium is an important element in the human body, amounting to about 50 mEq/kg. Ninety-eight percent of K^+^ is found within cells, while only 2% is in the extracellular fluid (8). There is evidence showing that elevated extracellular potassium characteristic of the extracellular space within tumors reduced the uptake and consumption of local nutrients by antitumor T cells (9). T cells in the tumor microenvironment are under metabolic constraints that dampen their activity and lead to cancer progression (10), indicating that high levels of potassium in the tumor microenvironment may suppress T-cell effector function. A cohort study also revealed that the level of fasting serum potassium in healthy men was positively associated with long-term cancer risk (11). Moreover, previous studies have claimed that hypokalemia is a major adverse event in the treatment of NSCLC that may provoke cardiac arrhythmias and/or respiratory arrest, thus requiring close monitoring and rapid correction (12, 13). In the immune system, the disorder of potassium homeostasis has been indicated as a determinant of immune dysfunction (8).

Therefore, we hypothesized that there would be an association between the level of serum potassium and the effect of targeted therapy on NSCLC patients. To verify the hypothesis, we conducted a meta-analysis to explore the relation between the efficacy of anti-EGFR therapy on NSCLC and the incidence of hypokalemia.

## Materials and Methods

### Search Strategy

A literature search was conducted in electronic datasets from PubMed, Embase, and Cochrane Library in April 2019 using the following various combinations of different keywords: “EGFR”, “epidermal growth factor receptor”, “monoclonal antibodies”, “tyrosine kinase inhibitors”, “cetuximab”, “gefitinib”, “erlotinib”, “icotinib”, “dacomitinib”, “afatinib”, “osimertinib”, “necitumumab”, “panitumumab”, “non-small cell lung cancer” “NSCLC”, and “hypokalemia”. The search was restricted to clinical trials published in English. The relevant reviews and meta-analyses were also examined for inclusive trials.

### Selection Criteria

Inclusion of relevant studies was based on the following criteria: 1) patients were pathologically confirmed to have stage III or IV NSCLC; 2) research studies were phase II/III prospective clinical trials; 3) all patients were administered anti-EGFR therapy alone or combined with other therapy; and 4) studies that reported the objective response rate (ORR) and/or disease control rate (DCR) and the exact number of patients with occurrences of hypokalemia of high grade (equal to or greater than grade 3).

### Data Extraction and Study Quality Assessment

Two reviewers independently reviewed the studies and reached consensus on all items. The following pieces of information were abstracted from the included studies: first author, publication year, country/region, phase of trial, line of treatment, intervention, number of patients, median age, sex ratio, ORR, DCR, and incidences of hypokalemia of grade ≥3. The study quality was independently assessed by the same two reviewers according to the Jadad score, which included randomization, blinding, and withdrawal, ranging from 0 to 5 points (14). Among all the included trials, the anti-EGFR monoclonal antibody or TKI treatment arms were included; otherwise, chemotherapy arms were collected for supplementary analysis. Placebo arms were excluded.

### Statistical Analysis

The ORR, DCR, and the incidence of hypokalemia of high grade (grade 3 or higher) were summarized from the data of all eligible trials. We calculated the proportions and 95% confidence intervals (CIs) of the ORR and DCR for each eligible trial. Heterogeneity among studies was evaluated using the Cochran’s *Q*-test and the *I* ^2^ statistics (15). The pooled proportions were calculated using a random effects model when the *p*-value <0.10 for the *Q*-test or the *I* ^2^ >50%. Otherwise, a fixed effects model was chosen. All *p*-values were two-tailed, and statistical significance was considered at *p* < 0.05. To determine whether the intervention type, line of treatment, trial phase, and drug numbers could represent potential sources of heterogeneity, subgroup analysis was performed. A sensitivity analysis was conducted with the pooled ORR/DCR re-calculated after excluding each trial at a time individually. All data analyses and the generation of forest plots were performed using R software (version 3.6.2).

## Results

**Figure 1:**
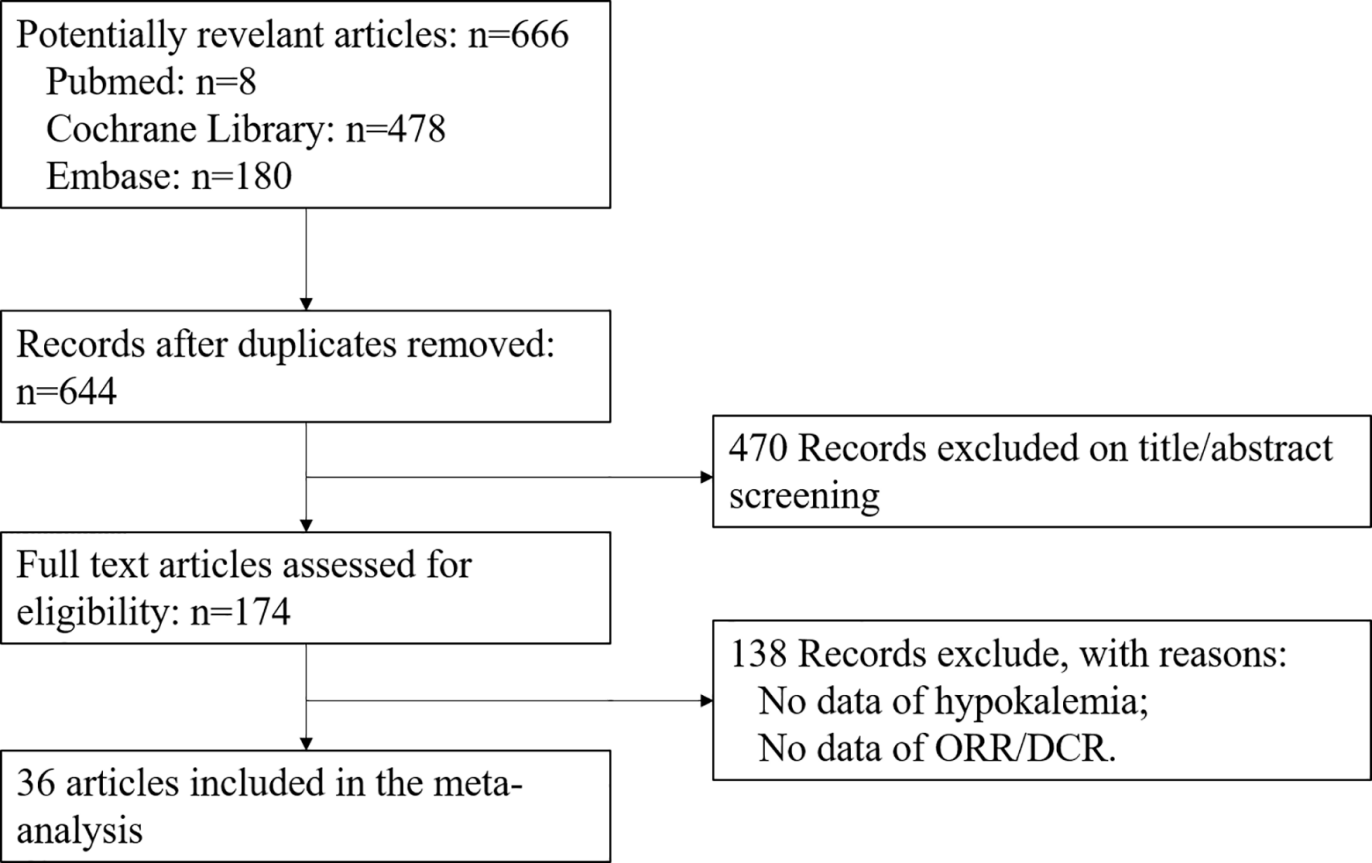
Outline of the literature search process.

**Figure 2:**
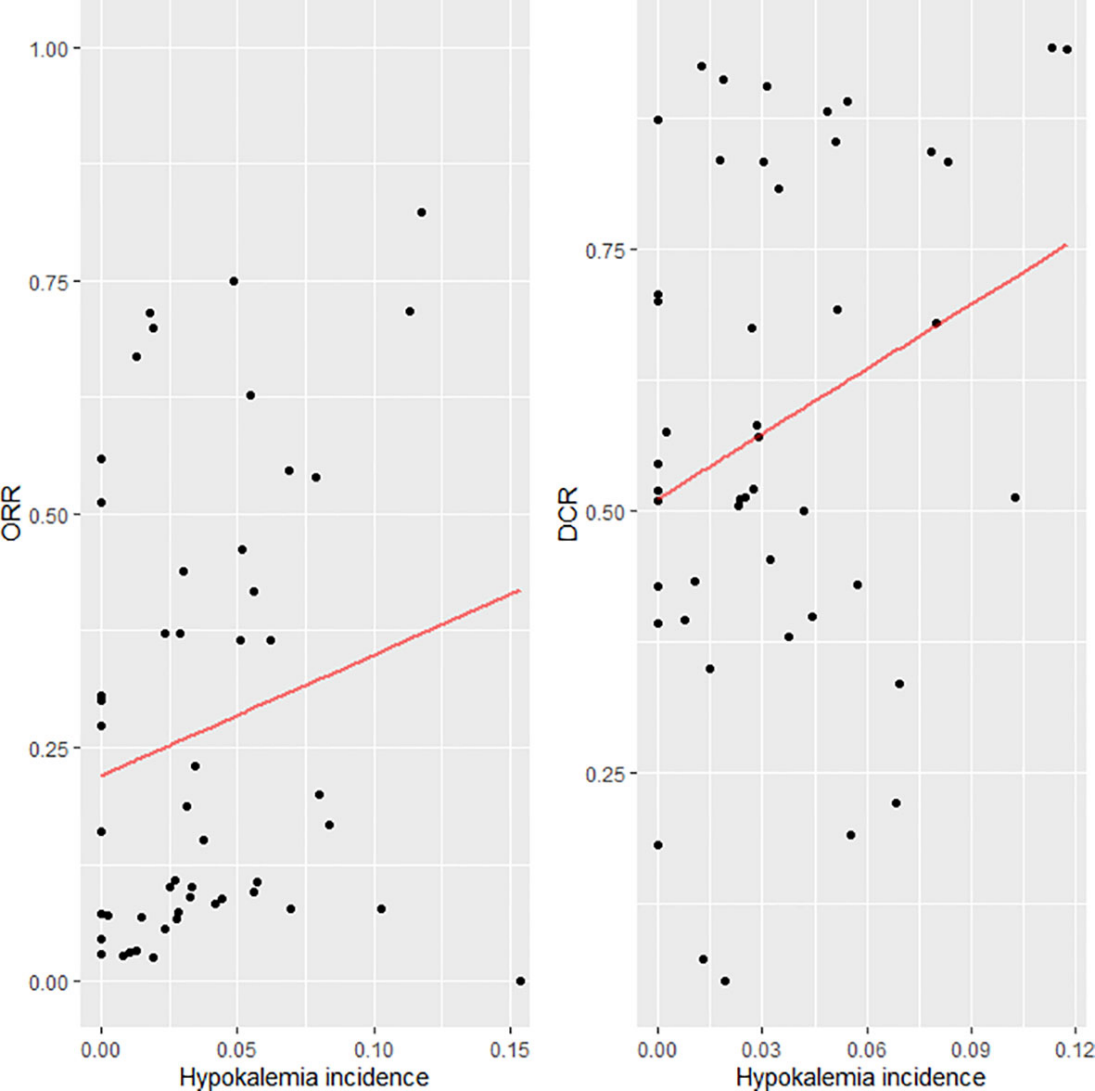
Scatter plot and fitted line for the incidence of hypokalemia and objective response rate (ORR)/disease control rate (DCR).

Of the 666 potentially relevant articles with anti-EGFR therapy screened, 36 clinical trials were finally included in this meta-analysis (**Figure 1**). Of these 36 studies, 15 were single-armed trials and 21 were randomized controlled trials. Eighteen studies used anti-EGFR treatment as first-line therapy and 18 did not. Treatment with anti-EGFR TKIs was evaluated in 26 studies, while 10 were studies on treatment with monoclonal antibodies. Twenty-five were phase II and 11 were phase III trials. A total of 9,761 patients were available for analysis. The characteristics of these trials are listed in **Table 1**. The relationship between hypokalemia incidence and ORR/DCR is scattered and fitted in **Figure 2**. A positive association could be observed in both scatter plots, except for an outlier in the lower right corner of the ORR plot. Except for the outlier, the highest hypokalemia incidence rate was 11.76%, with ORR of 82.35% and DCR of 94.12%. The lowest hypokalemia incidence rate was 0.00% in nine arms, with ORR ranging from 2.86% to 55.97% and DCR from 18.18% to 87.42%.

**Table 1:** Characteristics of the studies included in the meta-analysis.

First author	Year	Country/region	Trial design	Trial phase	Treatment line	Participants, *n*	Sex (male/female)	Age (years), median	Intervention	Intervention type	Efficacy	Jadad score
Niho et al. (16)	2006	Japan	SAT	II	FL	40	24/16	61	Gefitinib	TKI	ORR, DCR	–
Jackman et al. (17)	2007	–	SAT	II	FL	80	40/40	75	Erlotinib	TKI	ORR, DCR	–
Belani et al. (18)	2008	–	SAT	II	FL	80	42/38	63	Cetuximab+docetaxel+carboplatin	MA+other	ORR, DCR	–
Crino et al. (19)	2008	–	RCT	II	FL	97	75/22	74	Gefitinib	TKI	ORR, DCR	2
						99	73/26	74	Vinorelbine	other		
Lynch et al. (20)	2009	USA, Canada	RCT	II	NFL	25	11/14	62	Erlotinib+Bortezomib	TKI+other	ORR, DCR	3
					NFL	25	13/12	64	Erlotinib	TKI		
Pirker et al. (21)	2009	–	RCT	III	FL	557	385/172	59	Chemotherapy+cetuximab	MA+other	ORR	2
						568	405/163	60	Chemotherapy	other		
Govindan et al. (22)	2011	–	RCT	II	FL	53	24/19	66	Carboplatin+pemetrexed+cetuximab	MA+other	ORR, DCR	2
						48	27/21	65	Carboplatin+pemetrexed	other		
Ahn et al. (23)	2012	East Asia	RCT	II	FL	39	9/30	56	PC+gefitinib	TKI+other	ORR, DCR	3
						31	6/25	57	PC+pemetrexed	other		
Blumenschein et al. (24)	2012	–	SAT	II	NFL	30	18/12	64	sunitinib+erlotinib	TKI+other	ORR	–
Miller et al. (25)	2012	15 countries	RCT	IIB/III	NFL	390	159/231	58	Afatinib	TKI	ORR, DCR	5
						195	78/117	59	placebo	other		
Scagliotti et al. (26)	2012	–	RCT	III	NFL	480	297/183	61	sunitinib+erlotinib	TKI+other	ORR, DCR	5
					NFL	480	284/196	61	placebo+erlotinib	TKI+other		
Belani et al. (27)	2013	–	RCT	II	NFL	21	9/12	63	PF-3512676+erlotinib	TKI+other	ORR, DCR	2
					NFL	22	13/9	64	erlotinib	TKI		
Kim et al. (28)	2013	–	SAT	II	FL	102	52/50	64	Cetuximab+caboplatin+paclitoxel+ bevacizumab	MA+other	ORR, DCR	–
Kim et al. (29)	2013	Canada, USA	RCT	III	NFL	301	173/128	64	Premetrexed+cetuximab	MA+other	ORR, DCR	2
						304	188/116	65	Premetrexed	other		
					NFL	167	92/75	65	Docetaxel+cetuximab	MA+other	ORR, DCR	
						166	93/73	65	Docetaxel	other		
Ellis et al. (30)	2014	12 countries	RCT	III	NFL	480	244/236	63.5	Dacomitinib	TKI	ORR, DCR	5
						240	120/120	65.5	Placebo	other		
Janne et al. (31)	2014	China (Hong Kong), Japan, South Korea, China (Taiwan), USA	SAT	II	FL	89	29/60	62	Dacomitinib	TKI	ORR, DCR	–
Wu (32)	2014	China, Thailand, South Korea	RCT	III	FL	242	87/155	58	Afatinib	TKI	ORR, DCR	3
						122	39/83	58	Gemcitabine+cisplatin	other		
Han et al. (33)	2015	–	SAT	II	NFL	37	21/16	56	Gefitinib+vorinostat	TKI+other	ORR, DCR	–
Heigener et al. (34)	2015	–	SAT	IIIB	FL	157	116/41	–	Chemotherapy+cetuximab every 2 weeks	MA+other	ORR, DCR	–
						154	106/48	–	Chemotherapy +cetuximab weekly	MA+other		–
Lara et al. (35)	2015	–	SAT	II	NFL	45	14/31	64	Erlotinib+MK-2206	TKI+other	ORR, DCR	–
					NFL	35	15/20	63	Erlotinib+MK-2206	TKI+other	ORR, DCR	–
Lee et al. (36)	2015	East Asia	RCT	II	NFL	41	8/33	57	Pemetrexed+ erlotinib	TKI+other	ORR, DCR	1
					NFL	49	14/35	56.2	Erlotinib	TKI		
						43	15/28	54.8	Pemetrexed	other		
		Non-East Asia	RCT	II	NFL	37	12/25	55	Pemetrexed+erlotinib	TKI+other	ORR, DCR	1
					NFL	33	14/19	50.5	Erlotinib	TKI		
						37	20/17	57.6	Pemetrexed	other		
Liu et al. (37)	2015	–	SAT	I/II	FL	17	13/4	58	Cetuximab+inductive chemotherapy+chemoradiotherapy	MA+other	ORR, DCR	–
Wu et al. (38)	2015	China, Malaysia, Philippines	RCT	III	FL	110	42/68	57.5	Erlotinib	TKI	ORR, DCR	2
						107	42/65	56	Gemcitabine+cisplatin	other		
Lee et al. (39)	2016	–	RCT	II	NFL	25	11/14	63	Afatinib	TKI	ORR, DCR	1
					NFL	28	10/18	59	Erlotinib	TKI		
Park et al. (40)	2016	13 countries	RCT	IIB	FL	160	69/91	63	Afatinib	TKI	ORR, DCR	3
					FL	159	53/106	63	Gefitinib	TKI		
Han et al. (41)	2017	South Korea	SAT	II	NFL	39	10/29	62	Poziotinib	TKI	ORR, DCR	–
Spigel et al. (42)	2017	–	SAT	II	FL	66	27/39	65	Panitumumab+pemetrexed+carboplatin	MA+other	ORR, DCR	–
Spigel et al. (43)	2017	–	RCT	II	NFL	24	8/16	67	Erlotinib+sorafenib	TKI+other	ORR, DCR	2
						28	10/18	63	Sorafenib	other		
Thomas et al. (44)	2017	Germany, USA	RCT	II	FL	59	44/15	58	BTH1677+cetuximab+carboplatin+paclitaxel	MA+other	ORR, DCR	2
					FL	29	17/12	65	Cetuximab+carboplatin+paclitaxel	MA+other		
Wakelee et al. (45)	2017	–	RCT	II	NFL	13	6/7	64.8	Cabozantinib+erlotinib	TKI+other	ORR	1
						15	3/12	54.7	Cabozantinib	other		
Wu et al. (46)	2017	China, China (Hong Kong), Japan, South Korea, Poland, Italy, Spain	RCT	III	FL	227	81/146	62	Dacomitinib	TKI	ORR, DCR	3
					FL	225	100/125	61	Gefitinib	TKI		
Hata et al. (47)	2018	–	SAT	II	NFL	32	11/21	66	Afatinib+bevacizumab	TKI+other	ORR, DCR	–
Herbst et al. (48)	2018	–	RCT	III	FL	656	385/271	63	Chemotherapy+cetuximab	MA+other	ORR	3
						657	359/298	63	Chemotherapy	other		
Lu et al. (49)	2018	–	RCT	III	NFL	398	335/63	65	Afatinib	TKI	ORR, DCR	3
					NFL	397	331/66	64	Erlotinib	TKI		
Oda et al. (50)	2018	–	SAT	II	NFL	12	3/9	67.5	Afatinib	TKI	ORR, DCR	–
Reckamp et al. (51)	2019	–	SAT	II	NFL	37	14/23	64.6	Cabozantinib+erlotinib	TKI	ORR, DCR	–

RCT, randomized controlled trial; SAT, single-arm trial; FL, first line; NFL, not first line; MA, monoclonal antibodies; TKI, tyrosine kinase inhibitor; ORR, objective response rate; DCR, disease control rate.

**Figure 3:**
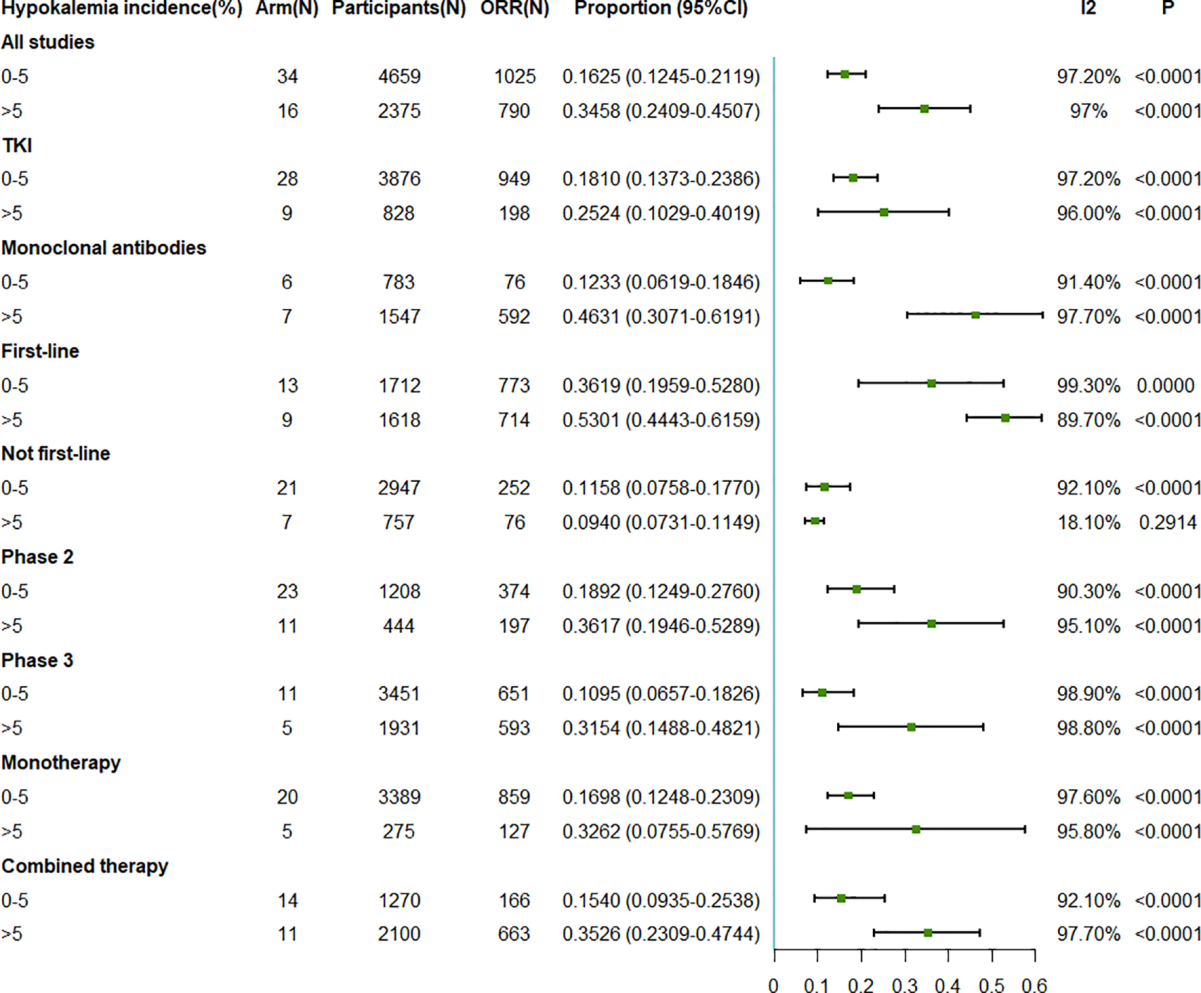
Forest plot for the meta-analysis of the ORR of anti-epidermal growth factor receptor (EGFR) targeted therapy for different incidence rates of grade 3–5 hypokalemia. *ORR*, objective response rate; *TKI*, tyrosine kinase inhibitor.

We observed that the pooled ORR was positively associated with the incidence of hypokalemia. The pooled ORR was 16.25% (95%CI = 12.45–21.19) when the incidence of hypokalemia was 0%–5%, while it increased to 34.58% (95%CI = 24.09–45.07) when the incidence of hypokalemia was greater than 5% (**Figure 3**). In the subgroup analysis on intervention type, the association was consistent. For TKI therapy, the pooled ORRs were 18.10% (95%CI = 13.73–23.86%) and 25.24% (95%CI = 10.29–40.19) when the hypokalemia incidence rates were ≤5% and >5%, respectively. Similar better ORRs with higher hypokalemia incidence rates could be observed in the monoclonal antibody treatment arms. As for the line of treatment, the pooled ORRs related to first-line treatment were 36.19% (95%CI = 19.59–52.80) and 53.01% (95%CI = 44.43–61.59) when the hypokalemia incidence rates were 0%–5% and >5%, respectively. However, for the other treatment types that were not first line, the ORRs were 11.58% (95%CI = 7.58–17.70) and 9.40% (95%CI = 7.31–11.49) when the hypokalemia incidence rates were 0%–5% and >5%, respectively. For the subgroup analysis on the different phases and drug numbers, the results were consistent with those of the whole population (**Figure 3**).

**Figure 4:**
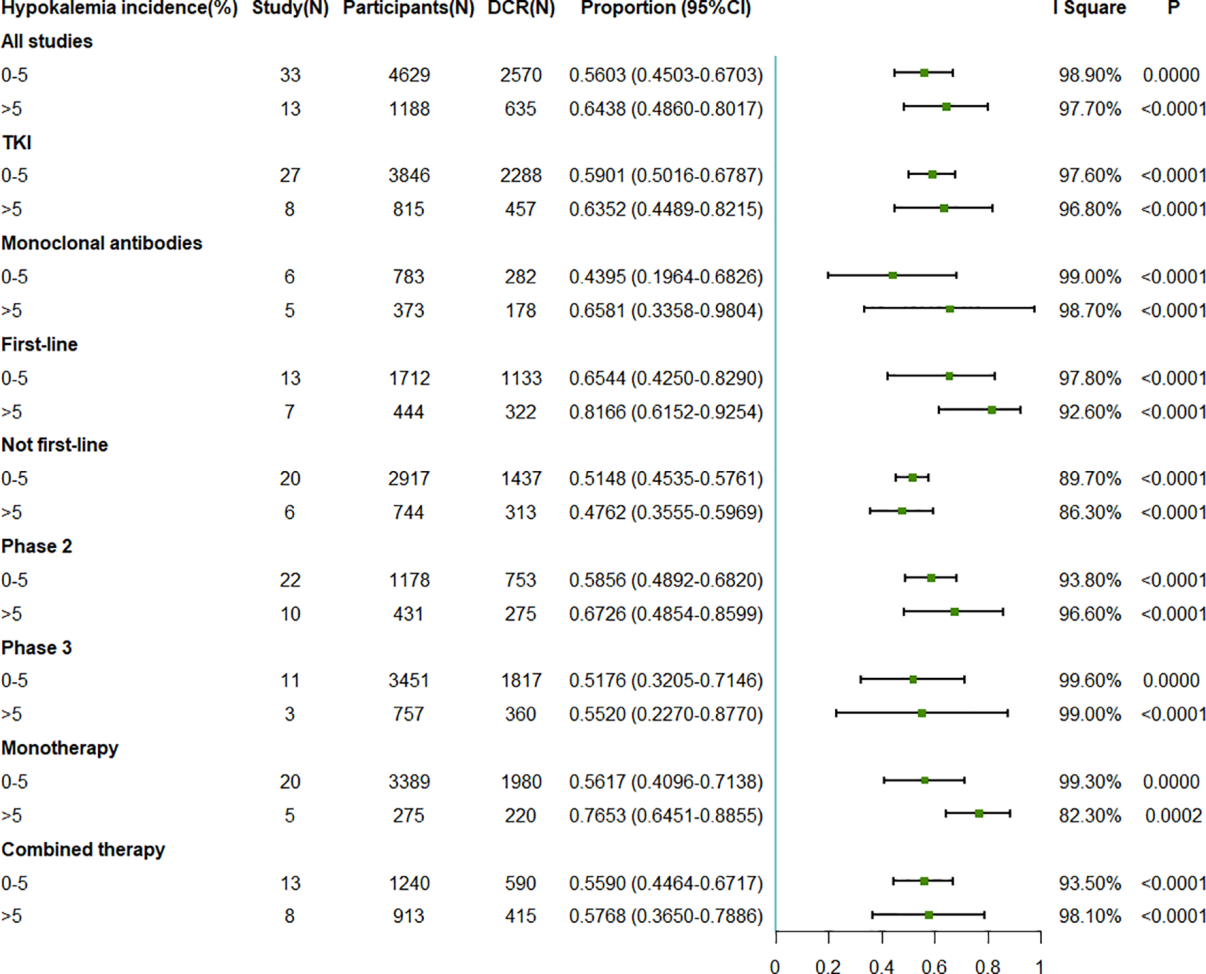
Forest plot for the meta-analysis of the DCR of anti-epidermal growth factor receptor (EGFR) targeted therapy for different incidence rates of grade 3–5 hypokalemia. *DCR*, disease control rate; *TKI*, tyrosine kinase inhibitor.

The pooled DCRs associated with EGFR antagonist were 56.03% (95%CI = 45.03–67.03) when the incidence of hypokalemia was 0%–5% and 64.38% (95%CI = 48.60–80.17) when the incidence of hypokalemia was >5% (**Figure 4**). In the subgroup analysis on the different intervention types, first-line treatment, different phases, and different drug numbers, the results were consistent with those observed in the whole population. However, similar to the ORR for the not first-line treatment, a higher DCR was observed with a lower hypokalemia incidence rate (**Figure 4**).

Sensitivity analysis showed non-obvious pooled ORR/DCR changes observed when excluding each trial at a time (**Supplementary Tables S1**, **S2**).

## Discussion

To the best of our knowledge, this is the first meta-analysis suggesting an association of an elevated incidence of hypokalemia with an increase in anti-EGFR treatment efficacy. The pooled ORRs were 16.25% and 34.58% and the pooled DCRs were 56.03% and 64.38% when the hypokalemia incidence rate ranges from ≤5% to >5%. These results indicated that the response to cancer therapy was associated with the serum potassium level.

In the carcinoma microenvironment, the concentrations of ions would be affected by high local levels of cellular apoptosis and necrosis. Potassium, as the most abundant intracellular ion, was significantly elevated 5–10 times in the tumor interstitial fluid compared with that in normal serum and benign tissue (52). Similarly, specific experimental apoptosis or necrosis was observed with the release of potassium into the extracellular microenvironment (52, 53). The elevated K^+^ acutely inhibited the T-cell receptor-induced production of effector cytokines, which resulted in subsequent immunosuppression (52). The elevated serum potassium limited the activity of antitumor T cells with metabolic constraints, eventually contributing to cancer progression (10). From this point of view, the hypokalemic microenvironment may strengthen the function of the immune system against tumor cells. The results were contradictory to the ORR and DCR (**Supplementary Tables S3**, **S4**) when considering the association between the effect of chemotherapy and the incidence of hypokalemia in our meta-analysis. Although a higher hypokalemia incidence was associated with a higher DCR, we observed inconsistent results for ORR. Thus, the association between cancer therapy and serum potassium level may be limited to targeted therapy. However, the exact mechanism between targeted therapy and hypokalemia is still unknown, and some researchers conjecture that this phenomenon may be due to the direct nephrotoxicity of targeted therapy (54, 55).

Overall, a higher incidence of hypokalemia was associated with better ORR and DCR. However, we observed an inverse association for the not first-line studies. In antitumor clinical trials, the possible causes of hypokalemia included drug nephrotoxicity and poor quality of life induced by the side effects of drugs, such as diarrhea, anorexia, and vomiting. A low serum potassium level enhanced the function of the immune system, making targeted therapies more effective. On the other hand, the better treatment effect of an anti-EGFR regimen with higher ORR and DCR may indicate longer survival of carcinoma patients, but possibly with worse quality of life. It is possible that the positive effect of hypokalemia on cancer treatment may be confounded by malnutrition with high loss and/or low potassium intake. From this viewpoint, the benefit from hypokalemia was offset by patients’ poor living conditions. To further explore our hypothesis on quality of life, the toxicity data and adverse event records of the 36 studies were collected. A higher incidence (6.7%) of diarrhea (grade ≥3) could be observed in the EGFR antagonist arm compared with that (1.3%) in other treatment arms (including chemotherapy, placebo, etc., data not shown). In the EGFR antagonist arm, there was a difference between the line of treatment and the incidence of diarrhea (grade ≥3), 3.94% and 9.17% for first line and not first line, respectively. Thus, it is possible that the change of serum potassium caused by diarrhea confounded the relationship between serum potassium level and treatment efficacy in the not first-line intervention. As for anorexia/decreased appetite (grade ≥3), weight loss/decreased weight (grade ≥3), and nausea/vomiting (grade ≥3), there were no obvious differences between the anti-EGFR arm and other treatment arms (data not shown). Also, in the EGFR antagonist arm, there was little difference between the different lines of treatment. Future mechanism research works and clinical trials are warranted to explore the effects of targeted therapy on serum potassium.

Previous studies have supported the association between lower serum potassium concentration and better outcomes in carcinoma, and more hypokalemia could be observed in targeted therapy. A Swedish perspective prostate cancer study, conducted with 11,492 participants, claimed that a weak positive association was observed between higher pre-diagnostic serum potassium (>5 mEq/L) and overall death (56). The Food and Drug Administration (FDA) review of panitumumab (Vectibix) for first-line use in metastatic colorectal cancer found that all grades of hypokalemia were observed with a 34% incidence rate and grades 3–5 with approximately 10% incidence rate. However, the incidence rates of hypokalemia in the non-panitumumab group were 14% and 4% for all grades and grades 3–5, respectively (54, 57). In a meta-analysis with a total of 2,254 participants, a higher incidence of grade 3 and 4 hypokalemia was positively associated with cetuximab-based therapy for advanced cancer (55). Similarly, when compared with non-cetuximab therapy, a higher risk of grade 3 and 4 hypokalemia with an odds ratio of 1.81 (95%CI = 1.12–2.93) was observed in the cetuximab arm (55). These studies, combined with our analysis, support a low serum potassium level as possibly beneficial for cancer patients in targeted therapy. Future studies are warranted to focus on how to maintain lower serum potassium levels to achieve better clinical outcomes.

However, hypokalemia, as an adverse event in cancer therapy, should be given sufficient attention for safety. Fluid and electrolyte imbalances were thought to be associated with increased mortality among hospitalized critically ill patients (56). In hospitalized cancer patients, hypokalemia is a common and important phenomenon, which may cause serious consequences such as cardiac arrhythmias and/or respiratory arrest. For outpatients, whose serum potassium levels were monitored even less closely than those of hospitalized ones, hypokalemia is also a dangerous adverse event (12, 55). Thus, monitoring of the serum potassium level in targeted therapy, even in cancer therapy, should be emphasized in this setting (13). Timely correction of modifiable clinical factors and management of electrolytes should not be ignored during the overall regimen period (58). The management of hypokalemia is based on strategies minimizing persistent losses and replacing serum potassium (54). Some research studies have revealed that the cause of hypokalemia is the compensation of serum magnesium deficiency (55, 59). Brief clinical check of blood magnesium ion concentrations is always warranted (60). Potassium replacement of a large amount should be gradually carried out, avoiding rebound hyperkalemia, until the clinical status of the cancer patient remains stable (54, 60). Thus, it is worth exploring how to keep a trade-off serum potassium level for both treatment effect and safety consideration to optimize prognosis.

Some limitations of our research are worth considering. Firstly, as a meta-analysis, the results were affected by the quality of each clinical trial. These included trials had different populations, follow-up durations, with or without chemotherapies, and different EGFR antagonists. The usage frequency of targeted therapy also varied among the trials, and some drugs were even only involved in a single clinical trial, e.g., “poziotinib”. Thus, detailed subgroup analysis for each anti-EGFR therapy was not possible. Moreover, only ORR and DCR were considered as the efficacy outcomes with different hypokalemia incidence levels, while time-to-event outcomes, such as overall survival and progression-free survival, are more important efficacy indexes in cancer therapy. Finally, it is impossible to obtain individual data for more detailed analysis to control for potential confounders.

In conclusion, our analysis has shown that the efficacy of anti-EGFR targeted therapy was associated with the incidence rate of hypokalemia. Compared with a hypokalemia incidence of 0%–5%, higher ORR and DCR could be observed with a hypokalemia incidence rate greater than 5%. Close monitoring and timely management of electrolytes should be emphasized in a carcinoma regimen, especially in targeted treatment. Different treatment effects should be considered for different serum potassium strata in future clinical trials with anti-EGFR therapy.

## Data Availability Statement

The original contributions presented in the study are included in the article/**Supplementary Material**. Further inquiries can be directed to the corresponding authors.

## Author Contributions

YZ, DY, TL, and JB were responsible for concept and design. JZ, YY, and XC acquired, analyzed, interpreted the data. JZ, YY, XC, and DY drafted the manuscript. YZ, JB, and DY critically revised the manuscript. All authors contributed to the article and approved the submitted version.

## Conflict of Interest

The authors declare that the research was conducted in the absence of any commercial or financial relationships that could be construed as a potential conflict of interest.

## Publisher’s Note

All claims expressed in this article are solely those of the authors and do not necessarily represent those of their affiliated organizations, or those of the publisher, the editors and the reviewers. Any product that may be evaluated in this article, or claim that may be made by its manufacturer, is not guaranteed or endorsed by the publisher.
