# Supplementary material for: Association of Hypokalemia Incidence and Better Treatment Response in NSCLC Patients: A Meta-Analysis and Systematic Review on Anti-EGFR Targeted Therapy Clinical Trials
Source: Front Oncol. 2022 Jan 5;11:757456. doi: 10.3389/fonc.2021.757456 (PMC8766730; doi:10.3389/fonc.2021.757456)
Supplement: Supplementary file 1 [file DataSheet_1.docx]

Supplementary Material

# Supplementary Tables

Supplementary table 1. Sensitivity analysis performed excluding each trial to test the stability of meta-analysis for pooled ORR (objective response rate) of anti-EGFR target therapy for different incidence of grade 3, 4, and 5 hypokalemia.

| Study excluded | | Arm  (N) | Hypokalemia incidence (%) | Proportion | (95%CI) | | I square | *P* |
| --- | --- | --- | --- | --- | --- | --- | --- | --- |
| Author | year |  |  |  |  |  |  |  |
| Niho | 2006 | 33 | 0-5 | 0.1590 | 0.1213 | 0.2085 | 97.27% | <0.0001 |
|  |  | 16 | >5 | 0.3458 | 0.2409 | 0.4507 | 97.32% | <0.0001 |
| Jackman | 2007 | 33 | 0-5 | 0.1652 | 0.1264 | 0.2160 | 97.22% | <0.0001 |
|  |  | 16 | >5 | 0.3458 | 0.2409 | 0.4507 | 97.32% | <0.0001 |
| Belani | 2008 | 33 | 0-5 | 0.1631 | 0.1246 | 0.2133 | 97.23% | <0.0001 |
|  |  | 16 | >5 | 0.3458 | 0.2409 | 0.4507 | 97.32% | <0.0001 |
| Crino | 2008 | 33 | 0-5 | 0.1691 | 0.1295 | 0.2207 | 97.22% | <0.0001 |
|  |  | 16 | >5 | 0.3458 | 0.2409 | 0.4507 | 97.32% | <0.0001 |
| Lynch | 2009 | 32 | 0-5 | 0.1646 | 0.1255 | 0.2158 | 97.34% | <0.0001 |
|  |  | 16 | >5 | 0.3458 | 0.2409 | 0.4507 | 97.32% | <0.0001 |
| Pirker | 2009 | 34 | 0-5 | 0.1625 | 0.1245 | 0.2119 | 97.20% | <0.0001 |
|  |  | 15 | >5 | 0.3449 | 0.2289 | 0.4610 | 97.31% | <0.0001 |
| Govindan | 2011 | 34 | 0-5 | 0.1625 | 0.1245 | 0.2119 | 97.20% | <0.0001 |
|  |  | 15 | >5 | 0.3209 | 0.2169 | 0.4250 | 97.21% | <0.0001 |
| Ahn | 2012 | 34 | 0-5 | 0.1625 | 0.1245 | 0.2119 | 97.20% | <0.0001 |
|  |  | 15 | >5 | 0.3386 | 0.2303 | 0.4469 | 97.47% | <0.0001 |
| Blumenschein | 2012 | 33 | 0-5 | 0.1644 | 0.1258 | 0.2150 | 97.26% | <0.0001 |
|  |  | 16 | >5 | 0.3458 | 0.2409 | 0.4507 | 97.32% | <0.0001 |
| Miller | 2012 | 33 | 0-5 | 0.1690 | 0.1304 | 0.2190 | 96.96% | <0.0001 |
|  |  | 16 | >5 | 0.3458 | 0.2409 | 0.4507 | 97.32% | <0.0001 |
| Scagliotti | 2012 | 33 | 0-5 | 0.1700 | 0.1315 | 0.2197 | 96.87% | <0.0001 |
|  |  | 15 | >5 | 0.3632 | 0.2517 | 0.4748 | 96.59% | <0.0001 |
| Belani | 2013 | 33 | 0-5 | 0.1652 | 0.1265 | 0.2158 | 97.27% | <0.0001 |
|  |  | 15 | >5 | 0.3623 | 0.2533 | 0.4714 | 97.47% | <0.0001 |
| Kim | 2013 | 34 | 0-5 | 0.1625 | 0.1245 | 0.2119 | 97.20% | <0.0001 |
|  |  | 15 | >5 | 0.3320 | 0.2250 | 0.4389 | 97.33% | <0.0001 |
| Kim | 2013 | 33 | 0-5 | 0.1690 | 0.1301 | 0.2196 | 97.04% | <0.0001 |
|  |  | 15 | >5 | 0.3652 | 0.2538 | 0.4766 | 97.09% | <0.0001 |
| Ellis | 2014 | 33 | 0-5 | 0.1698 | 0.1314 | 0.2195 | 96.87% | <0.0001 |
|  |  | 16 | >5 | 0.3458 | 0.2409 | 0.4507 | 97.32% | <0.0001 |
| Janne | 2014 | 34 | 0-5 | 0.1625 | 0.1245 | 0.2119 | 97.20% | <0.0001 |
|  |  | 15 | >5 | 0.3326 | 0.2254 | 0.4398 | 97.35% | <0.0001 |
| Wu | 2014 | 33 | 0-5 | 0.1509 | 0.1122 | 0.2030 | 97.23% | <0.0001 |
|  |  | 16 | >5 | 0.3458 | 0.2409 | 0.4507 | 97.32% | <0.0001 |
| Han | 2015 | 33 | 0-5 | 0.1576 | 0.1201 | 0.2068 | 97.28% | <0.0001 |
|  |  | 16 | >5 | 0.3458 | 0.2409 | 0.4507 | 97.32% | <0.0001 |
| Heigener | 2015 | 32 | 0-5 | 0.1795 | 0.1377 | 0.2340 | 97.18% | <0.0001 |
|  |  | 16 | >5 | 0.3458 | 0.2409 | 0.4507 | 97.32% | <0.0001 |
| Lara | 2015 | 32 | 0-5 | 0.1691 | 0.1292 | 0.2213 | 97.32% | <0.0001 |
|  |  | 16 | >5 | 0.3458 | 0.2409 | 0.4507 | 97.32% | <0.0001 |
| Lee | 2015 | 30 | 0-5 | 0.1432 | 0.1071 | 0.1914 | 97.50% | <0.0001 |
|  |  | 16 | >5 | 0.3458 | 0.2409 | 0.4507 | 97.32% | <0.0001 |
| Liu | 2015 | 34 | 0-5 | 0.1625 | 0.1245 | 0.2119 | 97.20% | <0.0001 |
|  |  | 15 | >5 | 0.3173 | 0.2123 | 0.4222 | 97.31% | <0.0001 |
| Wu | 2015 | 34 | 0-5 | 0.1625 | 0.1245 | 0.2119 | 97.20% | <0.0001 |
|  |  | 15 | >5 | 0.3260 | 0.2221 | 0.4300 | 97.14% | <0.0001 |
| Lee | 2016 | 33 | 0-5 | 0.1652 | 0.1264 | 0.2159 | 97.26% | <0.0001 |
|  |  | 15 | >5 | 0.3550 | 0.2460 | 0.4639 | 97.50% | <0.0001 |
| Park | 2016 | 32 | 0-5 | 0.1425 | 0.1043 | 0.1946 | 97.31% | <0.0001 |
|  |  | 16 | >5 | 0.3458 | 0.2409 | 0.4507 | 97.32% | <0.0001 |
| Han | 2017 | 34 | 0-5 | 0.1625 | 0.1245 | 0.2119 | 97.20% | <0.0001 |
|  |  | 15 | >5 | 0.3645 | 0.2549 | 0.4741 | 97.42% | <0.0001 |
| Spigel | 2017 | 33 | 0-5 | 0.1563 | 0.1189 | 0.2054 | 97.28% | <0.0001 |
|  |  | 16 | >5 | 0.3458 | 0.2409 | 0.4507 | 97.32% | <0.0001 |
| Spigel | 2017 | 33 | 0-5 | 0.1647 | 0.1260 | 0.2153 | 97.27% | <0.0001 |
|  |  | 16 | >5 | 0.3458 | 0.2409 | 0.4507 | 97.32% | <0.0001 |
| Thomas | 2017 | 33 | 0-5 | 0.1608 | 0.1228 | 0.2105 | 97.27% | <0.0001 |
|  |  | 15 | >5 | 0.3446 | 0.2357 | 0.4535 | 97.49% | <0.0001 |
| Wakelee | 2017 | 34 | 0-5 | 0.1625 | 0.1245 | 0.2119 | 97.20% | <0.0001 |
|  |  | 15 | >5 | 0.3694 | 0.2614 | 0.4775 | 97.38% | <0.0001 |
| Wu | 2017 | 32 | 0-5 | 0.1388 | 0.0991 | 0.1945 | 96.88% | <0.0001 |
|  |  | 16 | >5 | 0.3458 | 0.2409 | 0.4507 | 97.32% | <0.0001 |
| Hata | 2018 | 33 | 0-5 | 0.1618 | 0.1236 | 0.2119 | 97.26% | <0.0001 |
|  |  | 16 | >5 | 0.3458 | 0.2409 | 0.4507 | 97.32% | <0.0001 |
| Herbst | 2018 | 34 | 0-5 | 0.1625 | 0.1245 | 0.2119 | 97.20% | <0.0001 |
|  |  | 15 | >5 | 0.3409 | 0.2295 | 0.4523 | 97.04% | <0.0001 |
| Lu | 2018 | 32 | 0-5 | 0.1835 | 0.1425 | 0.2363 | 96.74% | <0.0001 |
|  |  | 16 | >5 | 0.3458 | 0.2409 | 0.4507 | 97.32% | <0.0001 |
| Oda | 2018 | 34 | 0-5 | 0.1625 | 0.1245 | 0.2119 | 97.20% | <0.0001 |
|  |  | 15 | >5 | 0.3559 | 0.2476 | 0.4642 | 97.50% | <0.0001 |
| Reckamp | 2019 | 33 | 0-5 | 0.1643 | 0.1256 | 0.2149 | 97.26% | <0.0001 |
|  |  | 16 | >5 | 0.3458 | 0.2409 | 0.4507 | 97.32% | <0.0001 |

Supplementary table 2. Sensitivity analysis performed excluding each trial to test the stability of meta-analysis for pooled DCR (disease control rate) of anti-EGFR target therapy for different incidence of grade 3, 4, and 5 hypokalemia.

| Study excluded | | Study excluded | Hypokalemia incidence (%) | Proportion | 95%CI | | I square | *P* |
| --- | --- | --- | --- | --- | --- | --- | --- | --- |
| Author | Author |  |  |  |  |  |  |  |
| Niho | 2006 | 32 | 0-5 | 0.5560 | 0.4440 | 0.6680 | 98.93% | <0.0001 |
|  |  | 13 | >5 | 0.6438 | 0.4860 | 0.8017 | 97.69% | <0.0001 |
| Jackman | 2007 | 32 | 0-5 | 0.5618 | 0.4494 | 0.6742 | 98.93% | <0.0001 |
|  |  | 13 | >5 | 0.6438 | 0.4860 | 0.8017 | 97.69% | <0.0001 |
| Belani | 2008 | 32 | 0-5 | 0.5660 | 0.4538 | 0.6782 | 98.93% | <0.0001 |
|  |  | 13 | >5 | 0.6438 | 0.4860 | 0.8017 | 97.69% | <0.0001 |
| Crino | 2008 | 32 | 0-5 | 0.5643 | 0.4519 | 0.6768 | 98.93% | <0.0001 |
|  |  | 13 | >5 | 0.6438 | 0.4860 | 0.8017 | 97.69% | <0.0001 |
| Lynch | 2009 | 31 | 0-5 | 0.5647 | 0.4510 | 0.6784 | 98.97% | <0.0001 |
|  |  | 13 | >5 | 0.6438 | 0.4860 | 0.8017 | 97.69% | <0.0001 |
| Govindan | 2011 | 33 | 0-5 | 0.5603 | 0.4503 | 0.6703 | 98.90% | <0.0001 |
|  |  | 12 | >5 | 0.6179 | 0.4585 | 0.7773 | 97.32% | <0.0001 |
| Ahn | 2012 | 33 | 0-5 | 0.5603 | 0.4503 | 0.6703 | 98.90% | <0.0001 |
|  |  | 12 | >5 | 0.6399 | 0.4730 | 0.8067 | 97.88% | <0.0001 |
| Miller | 2012 | 32 | 0-5 | 0.5596 | 0.4446 | 0.6745 | 98.93% | <0.0001 |
|  |  | 13 | >5 | 0.6438 | 0.4860 | 0.8017 | 97.69% | <0.0001 |
| Scagliotti | 2012 | 32 | 0-5 | 0.5671 | 0.4531 | 0.6810 | 98.89% | <0.0001 |
|  |  | 12 | >5 | 0.7083 | 0.5176 | 0.8461 | 94% | <0.0001 |
| Belani | 2013 | 32 | 0-5 | 0.5718 | 0.4602 | 0.6834 | 98.92% | <0.0001 |
|  |  | 12 | >5 | 0.6800 | 0.5182 | 0.8418 | 97.77% | <0.0001 |
| Kim | 2013 | 33 | 0-5 | 0.5603 | 0.4503 | 0.6703 | 98.90% | <0.0001 |
|  |  | 12 | >5 | 0.6802 | 0.5251 | 0.8354 | 97.37% | <0.0001 |
| Kim | 2013 | 32 | 0-5 | 0.5615 | 0.4474 | 0.6757 | 98.93% | <0.0001 |
|  |  | 12 | >5 | 0.6705 | 0.5078 | 0.8331 | 97.53% | <0.0001 |
| Ellis | 2014 | 32 | 0-5 | 0.5598 | 0.4441 | 0.6755 | 98.93% | <0.0001 |
|  |  | 13 | >5 | 0.6438 | 0.4860 | 0.8017 | 97.69% | <0.0001 |
| Janne | 2014 | 33 | 0-5 | 0.5603 | 0.4503 | 0.6703 | 98.90% | <0.0001 |
|  |  | 12 | >5 | 0.6267 | 0.4577 | 0.7957 | 97.75% | <0.0001 |
| Wu | 2014 | 32 | 0-5 | 0.5486 | 0.4408 | 0.6563 | 98.70% | <0.0001 |
|  |  | 13 | >5 | 0.6438 | 0.4860 | 0.8017 | 97.69% | <0.0001 |
| Han | 2015 | 32 | 0-5 | 0.5618 | 0.4498 | 0.6738 | 98.93% | <0.0001 |
|  |  | 13 | >5 | 0.6438 | 0.4860 | 0.8017 | 97.69% | <0.0001 |
| Heigener | 2015 | 31 | 0-5 | 0.6070 | 0.5264 | 0.6847 | 96.33% | <0.0001 |
|  |  | 13 | >5 | 0.6438 | 0.4860 | 0.8017 | 97.69% | <0.0001 |
| Lara | 2015 | 31 | 0-5 | 0.5695 | 0.4555 | 0.6835 | 98.96% | <0.0001 |
|  |  | 13 | >5 | 0.6438 | 0.4860 | 0.8017 | 97.69% | <0.0001 |
| Lee | 2015 | 29 | 0-5 | 0.5571 | 0.4385 | 0.6757 | 99.03% | <0.0001 |
|  |  | 13 | >5 | 0.6438 | 0.4860 | 0.8017 | 97.69% | <0.0001 |
| Liu | 2015 | 33 | 0-5 | 0.5603 | 0.4503 | 0.6703 | 98.90% | <0.0001 |
|  |  | 12 | >5 | 0.6188 | 0.4548 | 0.7829 | 97.76% | <0.0001 |
| Wu | 2015 | 33 | 0-5 | 0.5603 | 0.4503 | 0.6703 | 98.90% | <0.0001 |
|  |  | 12 | >5 | 0.6224 | 0.4573 | 0.7876 | 97.46% | <0.0001 |
| Lee | 2016 | 32 | 0-5 | 0.5653 | 0.4535 | 0.6771 | 98.93% | <0.0001 |
|  |  | 12 | >5 | 0.6410 | 0.4753 | 0.8067 | 97.88% | <0.0001 |
| Park | 2016 | 31 | 0-5 | 0.5381 | 0.4263 | 0.6500 | 98.79% | <0.0001 |
|  |  | 13 | >5 | 0.6438 | 0.4860 | 0.8017 | 97.69% | <0.0001 |
| Han | 2017 | 33 | 0-5 | 0.5603 | 0.4503 | 0.6703 | 98.90% | <0.0001 |
|  |  | 12 | >5 | 0.7015 | 0.5067 | 0.8432 | 94.45% | <0.0001 |
| Spigel | 2017 | 32 | 0-5 | 0.5516 | 0.4396 | 0.6637 | 98.92% | <0.0001 |
|  |  | 13 | >5 | 0.6438 | 0.4860 | 0.8017 | 97.69% | <0.0001 |
| Spigel | 2017 | 32 | 0-5 | 0.5621 | 0.4503 | 0.6739 | 98.93% | <0.0001 |
|  |  | 13 | >5 | 0.6438 | 0.4860 | 0.8017 | 97.69% | <0.0001 |
| Thomas | 2017 | 32 | 0-5 | 0.5528 | 0.4409 | 0.6646 | 98.93% | <0.0001 |
|  |  | 12 | >5 | 0.6262 | 0.4595 | 0.7928 | 97.82% | <0.0001 |
| Wu | 2017 | 31 | 0-5 | 0.5404 | 0.4269 | 0.6540 | 98.81% | <0.0001 |
|  |  | 13 | >5 | 0.6438 | 0.4860 | 0.8017 | 97.69% | <0.0001 |
| Hata | 2018 | 32 | 0-5 | 0.5494 | 0.4377 | 0.6611 | 98.92% | <0.0001 |
|  |  | 13 | >5 | 0.6438 | 0.4860 | 0.8017 | 97.69% | <0.0001 |
| Lu | 2018 | 31 | 0-5 | 0.5675 | 0.4481 | 0.6869 | 98.95% | <0.0001 |
|  |  | 13 | >5 | 0.6438 | 0.4860 | 0.8017 | 97.69% | <0.0001 |
| Oda | 2018 | 33 | 0-5 | 0.5603 | 0.4503 | 0.6703 | 98.90% | <0.0001 |
|  |  | 12 | >5 | 0.6294 | 0.4648 | 0.7941 | 97.87% | <0.0001 |
| Reckamp | 2019 | 32 | 0-5 | 0.5568 | 0.4448 | 0.6688 | 98.93% | <0.0001 |
|  |  | 13 | >5 | 0.6438 | 0.4860 | 0.8017 | 97.69% | <0.0001 |

Supplementary table 3. Meta-analysis for ORR (objective response rate) of chemotherapy for different incidence of grade 3, 4, and 5 hypokalemia.

| Hypokalemia incidence (%) | Study (N) | Participants (N) | ORR (N) | Proportion (95%CI) | I square | *P* |
| --- | --- | --- | --- | --- | --- | --- |
| 0-5 | 8 | 1882 | 475 | 0.2491 (0.1274-0.3707) | 98.30% | <0.0001 |
| >5 | 3 | 266 | 67 | 0.2157 (0.0782-0.3531) | 87.60% | 0.0003 |

Supplementary table 4. Meta-analysis for DCR (disease control rate) of chemotherapy for different incidence of grade 3, 4, and 5 hypokalemia.

| Hypokalemia incidence (%) | Study (N) | Participants (N) | DCR (N) | Proportion (95%CI) | I square | *P* |
| --- | --- | --- | --- | --- | --- | --- |
| 0-5 | 6 | 691 | 335 | 0.5924 (0.2867-0.8981) | 99.20% | <0.0001 |
| >5 | 3 | 266 | 198 | 0.7444 (0.6885-0.7932) | 39.80% | 0.1900 |
